# Bacterial Adherence Around Sutures of Different Material at Grafted Site: A Microbiological Analysis

**DOI:** 10.3390/ma12182848

**Published:** 2019-09-04

**Authors:** Lanka Mahesh, Varun Raj Kumar, Anshi Jain, Sagrika Shukla, Juan Manuel Aragoneses, José María Martínez González, Manuel Fernández-Domínguez, José Luis Calvo-Guirado

**Affiliations:** 1 Private Practice, New Delhi 110001, India; 2 Department of Oral Pathology, ITS Dental College, Ghaziabad 201009, Uttar Pradesh, India; 3 Department of Periodontology, All India Institute of Medical Sciences, Rishikesh 249201, India; 4 Department of Dental Research in Universidad Federico Henríquez y Carvajal (UFHEC), Santo Domingo 10107, Dominican Republic; 5 Department Oral Surgery, University Complutense of Madrid, 28223 Madrid, Spain; 6 Department of Oral Surgery and Implantology and Director of Research in Dentistry in the Doctoral Program of Translational Medicine, CEU San Pablo University, 28223 Madrid, Spain; 7 Department of Oral Surgery and Implant Dentistry, Faculty of Health Sciences, Universidad Católica de Murcia, UCAM, 30107 Murcia, Spain

**Keywords:** dental sutures, gut, silk, Vicryl, PTFE, polyamide

## Abstract

Closure of the surgical incision has been the primary function of sutures since their introduction. However, whatever the type, they are known to carry bacteria, which can be a source of infection. Five types of surgical sutures, Gut, Silk, Vicryl, PTFE, and Polyamide, were selected and tested on their ability to carry aerobic and anaerobic bacteria and were rated on the basis of forming colony-forming units (CFUs). Aerobic bacteria grown around gut sutures showed minimum CFUs (≈30 × 10^4^/suture). Though very less anaerobic bacteria growth was seen among all tested suture materials, it was maximum around Vicryl and polyamide sutures. Every suture material is capable, albeit not equally, of holding bacterial biofilm formation, which can be a source of surgical site infection.

## 1. Introduction

Since 3500 BC, sutures have been considered to be the most effective and useful method for the closure of surgical incisions and they have becomes an integral part of surgical procedures [1]. Surgical sutures are medical devices which are used to hold the open ends of the wounds together and should withstand physiologic mechanical stress, which help in wound closure. There are various types of sutures, such as absorbable/non-absorbable, synthetic/natural, monofilament/braided, etc., but all are known to attract bacteria, which can be a source of infection at the surgical site [1]. As is well known, superficial skin harbors bacterial colony at the time of operation. Therefore, during wound closure, procedure bacteria have the potential to move from the superficial skin surface into the deeper tissue layers carried by suture materials. It has been identified that suture material can act as a nidus for bacterial colonization and growth [2]. Bacterial accumulation at the surgical site changes and creates a hypoxic environment within and around the wound as well as inhibiting the activity of fibroblasts, which result in delayed healing [3]. Suture contamination by bacterial accumulation further leads to wound decontamination. The process of wound decontamination is mediated by the inhibition of the activity of granulocytic cell populations, which are required for providing defense activity in the human body [4]. However, the infection at surgical site is further aggravated by the formation of biofilm, where bacteria encapsulate themselves within a self-secreted extracellular polymeric slime matrix composed of polysaccharides, proteins, and nucleic acids [5,6]. In more than 60% of clinical infections, bacteria hide themselves in such biofilm and remain in a protected environment for a long time [7].

Suture materials with varied physical configuration and chemical structures are important for bacterial adhesion [8]. One of the most commonly known examples is braided sutures, which provide a larger and more complex surface for bacterial adherence than monofilament sutures, facilitating the entrapment of bacteria and thus increasing the risk of contamination [9,10].

The work conducted by Varma et al., Elek and Cohen, and Raju et al. elucidated that microbial burden is required to produce an infection in a clean surgical wound [11,12,13]. Recent reports by Kathju and colleagues suggested that the contamination of surgical sutures at the time of implantation by biofilm necessitates the eventual removal of the infective device [14,15]. Unlike other implantable devices which cause bacterial adherence, these surgical closure devices have not always been in the same limelight, though the surface characteristics of these devices make them a more susceptible substrate for bacterial adherence and/or contamination. When the wound microbial burden is low (2.0 log10 colony-forming units (CFUs)), the infection may present as a late-onset or chronic process that is nonresponsive to traditional therapeutic strategies [15,16]. While little research to date has been documented for the incidence of surgical site infections associated with contaminated surgical sutures, data from other implantable device-related infections suggest that these inert surfaces provide a hospitable niche for bacterial growth and proliferation. Many of these infections involve organisms which are resistant to antimicrobial therapy and host immune response, and are capable of producing a biofilm, thus allowing microbial persistence. Fewer data are available regarding suture site infection; therefore, this study conducted to investigate the microbial recovery from sutures explanted from non-infected or infected clinical specimens is one of a kind. Similar studies that have been conducted previously involved fewer than 10 patients and were limited to a selective surgical patient population [14,15].

## 2. Materials and Methods

**Figure 1:**
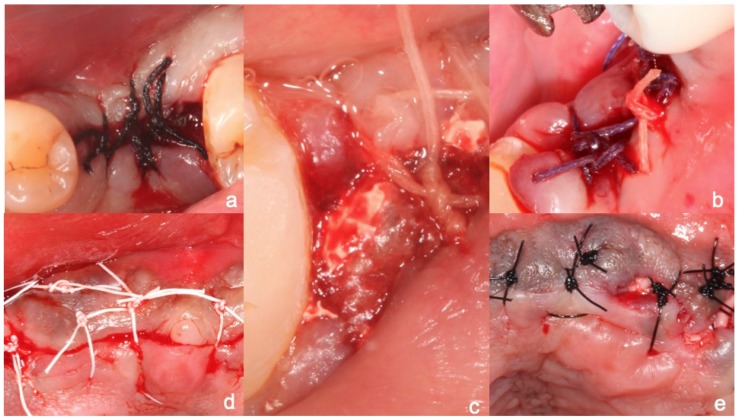
(**a**) Silk suture; (**b**) Vicryl: coated polyglactin; (**c**) Gut suture; (**d**) PTFE: synthetic fluoropolymer of tetrafluoroethylene; (**e**) polyamide suture.

Five types of sutures were selected for the study (Figure 1):

a) Silk suture: a nonabsorbable braided surgical suture obtained from cocoons of the silk worm and dyed black (Figure 1a).
b) Vicryl: polyglactin suture which is coated and composed of a copolymer made from 90% glycolide and 10% L-LACTIDE (Figure 1b).
c) Gut suture: strands of purified collagen taken from the serosal or submucosal layer of the small intestine of healthy ruminants (cattle, sheep, goats) or from beef tendon, which are twisted together. Catgut suture is a type of surgical suture that is naturally degraded by the body’s own proteolytic enzymes. Absorption is complete by 90 days, and full tensile strength remains for at least 7 days (Figure 1c).
d) PTFE: synthetic fluoropolymer of tetrafluoroethylene. These are hydrophobic, non-wicking monofilament sutures which reduce friction to a great extent. This property prevents bacteria from adhering to the surface (Figure 1d).
e) Polyamide suture material: a non-absorbable, monofilament suture whose fibers are tough, with high tensile strength, elasticity, and luster (Figure 1d).

### 2.1. Inclusion Criteria

(a) Patients scheduled for implant or surgery with guided bone regeneration (GBR) with a surgical incision site large enough to include at least four sutures with a distance of 4 mm between them.
(b) Patients willing to participate in the study, who filled in a signed consent form.
(c) Patients without known systemic illness (diabetes, heart disease, immune deficiency, thrombocytopenia/coagulation enzymes deficiency).
(d) Patients without the habit of chronic alcohol consumption/drug consumption or smoking.
(e) No pregnancy.

Data were recorded for every patient, which included age, gender, type of surgery, and postoperative care. Whether the patient was instructed to take antibiotics following surgery was also included.

A total of 50 patients were selected for the study that needed guided bone regeneration (GBR) for implant surgery. Thirty-two were male and 18 were female in the age group of 40–65 years. Suture segments were removed 14 days postoperatively according to standard protocol and under sterile conditions. Separate sterile forceps and scissors were used to remove each suture segment and transferred into a tube containing 1 mL of sterile phosphate buffer saline. The sutures were divided into five groups according to the suture, used with 10 specimens in each group. Once the samples were obtained, they were immediately transferred to the laboratory for microbiological analysis.

### 2.2. Bacterial Culture

Before inoculating the samples on culture media, vortexing was done for 10 and a series of four 10-fold dilutions of each sample were prepared. Ten milliliters of each dilution were seeded on two blood agar plates and labeled. One plate of each sample was incubated for 24–48 h at 37 °C under aerobic conditions for aerobic bacteria and yeasts, and under 5% to 10% carbon dioxide (CO_2_) atmosphere for aerobic/anaerobic facultative bacteria. A similar identical plate was incubated 48–72 h at 37 °C under strictly anaerobic conditions within an anaerobic system (N_2_ 85%, H_2_ 5%, CO_2_ 10%) using an anaerobic gas jar and gas pack for anaerobe obligate and facultative bacteria. Following the incubation period, a colony counter was used to count the colonies and to calculate the number of colony-forming units (CFUs). The number of black-pigmented CFUs was counted on plates both from aerobic and anaerobic conditions. A CFU was calculated as the number of colonies per 10 μL at the minimum dilution with clear, separate, and countable colonies.

## 3. Results

**Figure 2:**
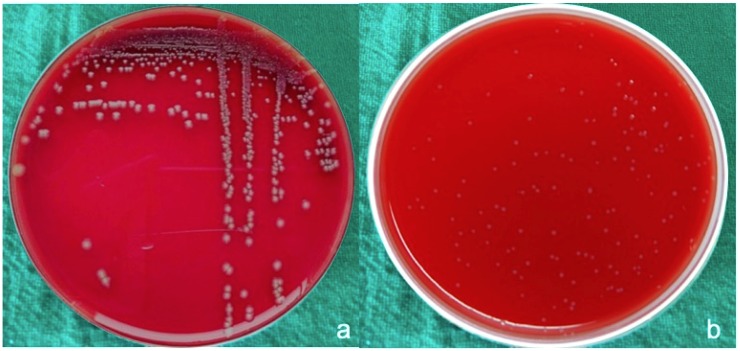
(**a**) Aerobic bacteria grown around gut sutures, (**b**) anaerobic bacteria grown around gut sutures.

**Figure 3:**
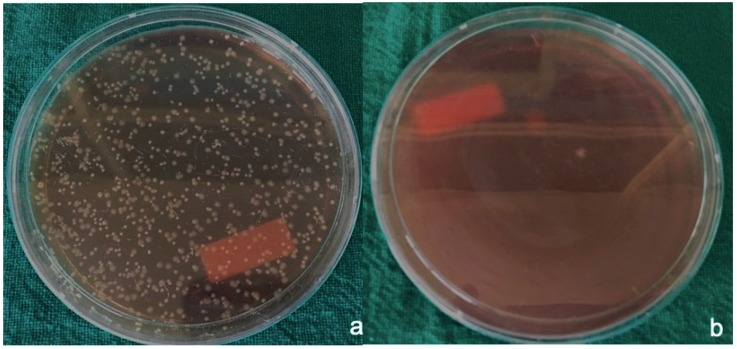
(**a**) Aerobic bacteria grown around silk sutures, (**b**) anaerobic bacteria grown around silk sutures.

Results were expressed as the mean CFUs (± standard deviation) of total bacteria for both anaerobic bacteria and aerobic bacteria. Aerobic bacteria grew around gut sutures (Figure 2a) and showed minimum CFUs (≈30 × 10^4^/suture) in comparison to other sutures, followed by silk sutures (Figure 3a) at a CFU total of approximately 102 × 10^4^/suture.

**Figure 4:**
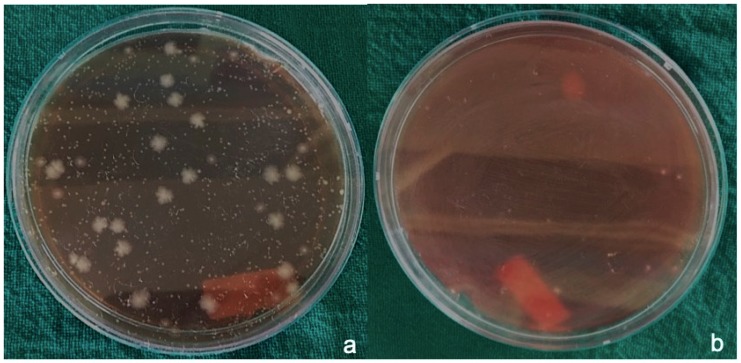
(**a**) Aerobic bacteria grown around PTFE sutures, (**b**) anaerobic bacteria grown around PTFE sutures.

**Figure 5:**
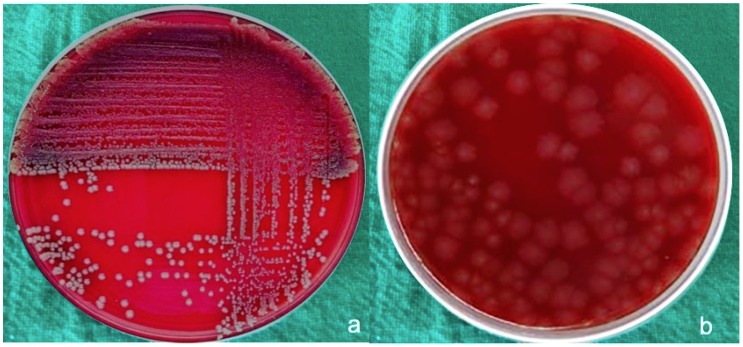
(**a**) Aerobic bacteria grown around polyamide sutures, (**b**) anaerobic bacteria grown around polyamide sutures.

**Figure 6:**
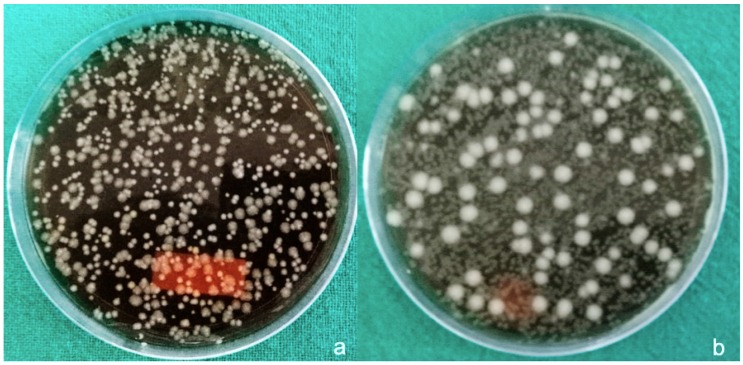
(**a**) Aerobic bacteria grown around Vicryl sutures, (**b**) anaerobic bacteria grown around Vicryl sutures.

PTFE (Figure 4a) and polyamide sutures (Figure 5a) showed almost equal amounts of CFUs (≈300 × 10^4^/suture) and vicryl sutures (Figure 6a) showed the maximum growth of aerobically cultured bacteria CFUs (≈444 × 10^4^/suture).

Much less growth of anaerobic bacteria was seen around the suture material used in the study, with CFU values of 10 × 10^4^/suture around gut sutures (Figure 2b) and silk sutures (Figure 3b). Little variation was observed around PTFE sutures (Figure 4b) with CFU values of 20 × 10^4^/suture. Polyamide (Figure 5b) and Vicryl sutures (Figure 6b) showed the maximum anaerobic growth among all the suture materials used in the study, with CFU values of 50 × 10^4^/suture.

Results were analyzed statistically using the ANOVA-t test, which showed that the difference between aerobic and anaerobic bacterial adherence around different suture materials within the group was statistically significant.

Further observation was also conducted between the study groups (different suture materials) by analyzing the bacterial load. The obtained results were statistically significant and showed that gut sutures have minimum adherence of aerobic and anaerobic bacteria in comparison to other study groups (Table 1).

## 4. Discussion

The current study stated that the suture materials plays an intimate role in the adhesion and growth of varieties of bacteria as well as the formation of biofilm. Overall, the adhesion of bacteria to gut sutures was found to be lower compared to silk, PTFE, polyamide, and Vicryl sutures. The flow of bacteria from the superficial surface of the oral environment, along the suture canal, and deep into tissues can evoke an inflammatory response [17]. The medical and dental research literature stresses the harmful effect of bacterial adhesion to the surface of suture materials, and it indicates a clear advantage of monofilament non-resorbable sutures [18]. One very interesting statement discussed and put forth by Edlich et al. is that the chemical structure of the suture was found to be the most important factor in the development of surgical infection rather the physical configuration, which played a relatively minor role in surgical site infection. However, the findings of our study are not in agreement with these results, as less accumulation of bacteria was seen around silk sutures in comparison to Vicryl sutures [19]. Another important parameter analyzed in this study was the presence of anerobic and anaerobic bacterial accumulation around the suture material. The results revealed less anaerobic in comparison to aerobic bacteria. A probable hypothesis explaining this result is that, supra gingivally, more aerobic bacteria are seen, whereas anaerobic bacteria are predominant in subgingival plaque [20]. Our results strongly indicate that, whenever possible, the first choice of suture between the present tested materials should be gut suture. If gut sutures cannot be used, the selection between the other tested suture materials may be subject to the personal preference of the surgeon. Furthermore, since all sutures were found to harbor bacteria and support their accumulation, any suture may act as a port for the entry of infection, which may compromise the surgical wound healing. Therefore, it is advised to minimize the use of sutures at the surgical site. Moreover, their removal should be carried out as early as possible, according to the specific healing conditions [21].

## 5. Conclusions

Surgical site infection is a common problem encountered after every surgical intervention. The formation of bacterial biofilm by attachment to the underlying foreign body or to the tissue substratum is another complication of any surgery. Hence, this study investigated different suture materials used at surgical sites and their contribution to surgical site infection. It was shown that any suture material can host or harbor bacterial biofilm formation, but not all suture materials are necessarily equivalent in this regard. The formation of the biofilm depends on the nature and type of the suture material, which should be carefully taken into consideration for implanted surgical sites. In the limited scope of this study, two types of suture, one monofilament (polyamide) and one braided (Vicryl), were found to harbor the maximum number of anaerobic bacteria. Therefore, these sutures may be detrimental to wound healing. Further studies are required to corroborate this evidence.

## Figures and Tables

**Table 1:** Bacterial load around different suture materials.

Type of Suture Material	Aerobic Bacterial Growth (CFUs)	Anaerobic Bacterial Growth (CFUs)	*p* Value
Gut sutures	≈30 × 10^4^/suture	10 × 10^4^/suture	<0.05
Silk sutures	≈102 × 10^4^/suture	10 × 10^4^/suture	
PTFE sutures	≈300 × 10^4^/suture	20 × 10^4^/suture	
Polyamide sutures	≈300 × 10^4^/suture	20 × 10^4^/suture	
Vicryl sutures	444 × 10^4^/suture	50 × 10^4^/suture	
*p* Value	<0.05		
